# Overview of Current Regulatory and Methodological Approaches for the Risk Assessment of Mycotoxins

**DOI:** 10.3390/toxins18070294

**Published:** 2026-07-05

**Authors:** Bozidar Udovicki, Dragan Milicevic, Andreja Rajkovic

**Affiliations:** 1 Department of Food Safety and Quality Management, Faculty of Agriculture, University of Belgrade, Nemanjina 6, Zemun, 11080 Belgrade, Serbia; andreja.rajkovic@ugent.be; 2 Institute of Meat Hygiene and Technology, Kaćanskog 13, 11000 Belgrade, Serbia; dragan.milicevic@inmes.rs; 3 Laboratory of Food Microbiology and Food Preservation, Department of Food Technology, Safety and Health, Faculty of Bioscience Engineering, Ghent University, Coupure Links 635, 9000 Ghent, Belgium

**Keywords:** mycotoxins, risk assessment, hazard assessment, exposure assessment, hazard characterization

## Abstract

Risk assessment is a dynamic and continuously evolving process aimed at characterizing the potential adverse effects on life and health arising from exposure to hazards. Among these hazards, mycotoxins represent one of the most significant and widely investigated contaminants in food and feed. This review aims to summarize current knowledge on mycotoxin risk assessment practice, both for single substances and for cumulative risk assessment, and to provide practical guidance for future researchers. As individual substances, mycotoxins are well characterized from a toxicological perspective, and numerous risk assessments have been conducted globally across a wide range of food products. Although well-established methodological frameworks exist to support the cumulative risk assessment of mycotoxins, further efforts are needed to define common assessment groups, especially considering the pleiotropic nature of these compounds.

## 1. Introduction

Food safety is a basic public health concern, and as a fundamental part of risk analysis, risk assessment is globally recognized as a core principle in establishing food policies [1,2,3]. Risk assessment, a process of characterizing the potential adverse effects on life and health arising from exposure to hazards over a specified period of time, represents a central scientific component of risk analysis and has evolved largely in response to the need to support decision-making pointed at protecting human health under conditions of scientific uncertainty [4]. It is a scientifically based process that includes four sequential steps: hazard identification, hazard characterization, exposure assessment, and risk characterization, which together support the evaluation of potential health risks. Risk assessment is a dynamic and continuously evolving process that adapts to advances in analytical methods, changes in agricultural practice and climate, increasing volume and diversity of food trade, increased public awareness and changes in dietary habits [4]. Continuous risk assessment provides valuable information on exposure trends, the effectiveness of control measures, and the impact of new agricultural, safety or food production practices [5]. Usually, regulatory approaches for assessing chemicals evaluate chemicals one at the time, an approach that fails to consider the human real-world scenario of low-dose combined exposures to chemical mixture [6]. Bearing in mind that humans are exposed to a contaminant’s mixture, in recent years, there has been a regulatory and scientific shift toward developing cumulative risk assessment models, emphasizing the growing complexity of the food safety environment and food safety risk assessment and the need for continuous improvement in toxicology assessments and methodological approaches.

Given their acute and chronic toxicity and their unavoidable presence in food, mycotoxins represent one of the most significant and widely investigated contaminants in food and feed, occurring globally. Of the approximately 400 different mycotoxins that have been identified to date, a relatively small group represents priority mycotoxins [7,8]. The most important mycotoxins affecting food safety are produced by filamentous fungi, mainly belonging to the genera *Aspergillus*, *Fusarium*, *Penicillium*, and to a minor extent *Alternaria* and *Claviceps* which encompass species responsible for key toxins including aflatoxins (AFs), fumonisins (FUMs), ochratoxin A (OTA), trichothecenes, zearalenone (ZEN), patulin (PAT), ergot alkaloids (EAs), and citrinin (CIT) [9,10]. Most important mycotoxins and their main producers are shown in Table 1. Fungal infection and the production of mycotoxins occur throughout the whole food chain, beginning in the field during plant growth, and may continue through harvesting, storage, and processing [10]. Mycotoxins can occur in a wide range of food commodities and, owing to their chemical stability, may also persist in highly processed foods. Of particular importance is their occurrence in staple crops, especially cereals. Furthermore, they often co-occur in crops, and given the diversity of modern diets, simultaneous exposure to mixtures of mycotoxins is common [11]. Co-occurrence with at least two mycotoxins was reported in over 50% of European cereals [7]. It is estimated that around 60–80% of global crops have detectable levels of mycotoxin, with a quarter of analyzed samples exceeding legal limits [11]. This is explained by a combination of the improved analytical sensitivity and the impact of climate change [11]. It is recognized that climate change will increase the complexity of mycotoxin risk as it may induce a shift in both geographical distribution and patterns of mycotoxin production [9,12].

Considering that mycotoxins rank among the most prevalent and important chemical contaminants in food, due to their toxic effects and frequent occurrence in food, they are strictly regulated globally. In the European Union, Maximum levels (MLs) for mycotoxins in food are set in Commission Regulation (EU) 2023/915 [21], which repealed long-standing Commission Regulation (EC) No. 1881/2006 [22]. While the final decision-making for setting MLs is determined through risk management processes, the scientific risk basis for the establishment and revision of MLs in food is grounded in scientific risk assessment conducted by the European Food Safety Authority (EFSA). The general recommendation for mycotoxins is to keep their levels “as low as reasonably achievable”—i.e., to the lowest level of contamination that can be reasonably achieved without removing the food from the food supply [23]. A clear example of the dynamic and continuously evolving nature of both the food safety ecosystem and risk assessment process is reflected in the growth of EU legislation, particularly in the transition from Commission Regulation (EC) No. 1881/2006 to the current Regulation (EU) 2023/915, as reflected in updated ML for established mycotoxins and the inclusion of newly regulated ones. The initial legal framework covered a partial number of major mycotoxins and included several dozen specific food-mycotoxin combinations with established MLs, while, in contrast, the current regulation expands both the range of regulated mycotoxins and the number of ML entries, now comprising over one hundred food-mycotoxin combinations [21,22]. It is reasonable to assume that the list of food–mycotoxin combinations with established MLs will continue to evolve in the coming years, as climate change represents an important risk factor that further emphasizes the need for continuous risk assessment.

This review aims to summarize current knowledge on mycotoxin risk assessment practice, focusing on methodological developments, for single substances and for cumulative risk assessment, and to provide practical guidance for future researchers.

## 2. Hazard Assessment

Hazard identification and hazard characterization are closely connected, frequently parallel processes in risk assessment, often referred to as hazard assessment [24]. The purpose of hazard identification is to evaluate the weight of evidence for adverse health effects, based on assessment of all available data on toxicity and mode of action (MoA), while hazard characterization describes the relationship between the administered dose of, or exposure to, a chemical and the incidence of an adverse health effect [25]. In other words, hazard identification aims to identify potential critical endpoints that may be relevant to human health, while hazard characterization explores the nature of dose–response relationships in detail, with the overall aim of identifying a dose from toxicity studies that will then be used to establish a level of human intake at which it is confidently expected that there would be no appreciable adverse health effects, taking into account uncertainty and variability [26].

### 2.1. Reference Points and Health-Based Guidance Values

The data obtained from toxicity studies may be further scrutinized to identify a dose (known as Reference Point (RP) or Point of Departure (PoD)) that can be used as a starting point in the establishment of health-based guidance values (HBGV) such as Tolerable Daily Intake (TDI) or Tolerable Weekly Intake (TWI), or used further in the risk assessment process [25,26]. Two main statistical approaches are currently used to establish an RP: the no-observed-adverse-effect level (NOAEL) approach and the benchmark dose (BMD) approach. NOAEL is defined as the highest dose tested in a study that does not result in observable adverse effects, while the LOAEL corresponds to the lowest dose at which a statistically significant adverse effect is detected [26]. The BMD is a dose level estimated from a fitted dose–response curve, corresponding to a specified change in response comparative to the control (background) response, known as the benchmark response [26]. The lower confidence bound of the BMD, referred to as the BMDL, is typically used as the RP.

Liver carcinogenicity, i.e., the development of hepatocellular carcinoma (HCC), is the critical effect of oral exposure to AFs. In the early AF exposure and risk assessment, performed to assess the impact of a possible change in the maximum levels for almonds, hazelnuts, and pistachios, the EFSA Panel on Contaminants in the Food Chain (CONTAM Panel) used a BMDL_10_ value of 0.17 μg/kg bw per day, from the range of 0.17 μg to 0.34 μg/kg bw per day, based on data from a study on male Fisher rats by Wogan et al. [27,28]. Another (human) BMDL_10_ of 0.87 μg/kg bw per day, based on the study of a population [29], was used for the risk characterization. These values were subsequently commonly found to be used in scientific literature regarding AF risk assessment. In 2020, the CONTAM Panel selected a BMDL_10_ of 0.4 μg/kg bw per day, using model averaging and using the same data for the incidence of HCC in male rats from the study of Wogan et al. [13,27]. In the same opinion, they concluded that the calculation of a BMDL from the human data was not appropriate.

In 2006, the EFSA CONTAM panel adopted a scientific opinion regarding OTA in food, concluding that it is a potent renal toxin that accumulates in the kidney. Based on the LOAEL of 8 μg/kg bw per day for the early markers of renal toxicity in pigs, they established a TWI of 0.12 μg/kg bw [30]. In its 2020 updated opinion on OTA, EFSA concluded that, in light of recent studies raising uncertainty regarding the MoA for kidney carcinogenicity, it is inappropriate to establish an HBGV [14]. For the characterization of non-neoplastic effects, a BMDL_10_ of 4.73 μg/kg bw per day was calculated based on the microscopic kidney lesions seen in the study of Krogh et al. [31] and used as a non-neoplastic RP [14]. For characterization of neoplastic effects, a BMDL_10_ of 14.5 μg/kg bw per day was calculated from the combined incidences of carcinoma and adenoma from the 2 year male rat oral study [32] and used as a neoplastic RP [14].

In 2014, EFSA evaluated the potential public health implications of a temporary derogation from the MLs for DON, ZEN, and FUMs in maize and maize products [33]. As it was not possible to carry out a full FUMs hazard characterization for this assessment, EFSA used the group provisional maximum TDI of 2 μg/kg bw established by the Joint FAO/WHO Expert Committee on Food Additives (JECFA) [34]. This value was reaffirmed in 2017, when JECFA published an additional assessment on FUMs [35]. In 2018, the CONTAM Panel derived a BMDL_10_ of 100 μg/kg of FB1 per day based on the increased incidence of megalocytic hepatocytes in the liver, which was considered the critical effect [36]. Based on this BMDL_10_, a TDI of 1 μg/kg bw per day for the FB1 was set [36]. Considering their structural similarity and the limited data suggesting similar MoA and toxic potency, the CONTAM Panel determined that FB2, FB3, and FB4 should be included with FB1 within a group TDI [36].

In a scientific report on the occurrence and exposure of DON, EFSA used a provisional TDI of 1 µg/kg bw per day, set in 2002 by the Scientific Committee for Food, based on a NOAEL of 100 μg/kg bw per day due to decreased body weight gain observed in a 2-year mice feeding study [37]. This value was confirmed in 2010 by JECFA, which extended it to the group of DON and its acetyl derivatives (3-acetyl-DON (3-Ac-DON) and 15-acetyl-DON) [37]. In a subsequent opinion, the CONTAM Panel identified reduced body weight gain in experimental animals as the critical endpoint for chronic human risk assessment, and using a BMDL_05_ of 110 μg/kg bw per day, the Panel established a group TDI of 1 μg/kg bw per day for the combined exposure to DON, 3-Ac-DON, 15-Ac-DON, and DON-3-glucoside [38].

In its initial assessment of T-2 and HT-2 toxins, the CONTAM Panel considered a reduced specific antibody response in pigs to be the critical endpoint for human risk assessment and, based on a BMDL_05_ of 10 μg T-2 toxin/kg bw per day, established a group TDI of 0.1 μg/kg bw per day for the combined exposure to T-2 and HT-2 toxins [39]. In the following 2017 evaluation, reduction in total leukocyte count was identified as the critical effect for T-2 and HT-2 toxins. Using this endpoint, a BMDL_10_ of 3.33 μg T2/kg bw per day was calculated, and a new group TDI for T-2 and HT-2 of 0.02 μg/kg bw was established [40].

In an earlier opinion on the public health risks associated with the presence of ZEN in food, a TDI of 0.25 μg/kg bw per day was derived based on the NOAEL of 10 μg/kg bw per day, with its estrogenic activity identified as the critical effect [16]. To date, no new studies have been identified to replace this TDI. In 2016, the CONTAM Panel established a group TDI of 0.25 μg/kg bw per day, expressed as ZEN equivalents, for ZEN and its modified forms. [41].

The provisional TDI for patulin is set at 0.4 µg/kg bw per day based on the no observable effect level of 43 µg/kg bw per day [42]. For EAs, the critical effect used for hazard characterization was the vasoconstrictive effect of ergotamine, observed through tail muscular atrophy in rats [18]. BMDL_10_ of 330 µg/kg bw per day was calculated, and a TDI of 0.6 μg/kg bw per day was established [18]. CIT was characterized based on the available data on nephrotoxicity. NOAEL of 20 µg/kg bw per day was used to determine a level of no concern for nephrotoxicity of a 0.2 µg/kg bw per day [19].

### 2.2. Combined Effects

Hazard assessment of combined exposure to multiple chemicals aims to derive quantitative metrics reflecting the combined toxicity of a certain mixture [24]. Chemical mixtures can be assessed with a whole-mixture approach when data on the mixture components are not available, or with a component-based approach when data on the mixture components are sufficient. In the whole mixture approach, the mixture is treated like a single chemical substance, while a component-based exposure assessment accounts for the variability of the mixture’s composition [24]. A component-based approach includes the grouping of chemicals into common assessment groups. These assessment groups can be formed based on regulatory criteria, on occurrence in a common source, on similarity of chemical structures (common functional groups, similar base structure, or similar carbon range numbers) and based on the compounds’ biological and toxicological properties [24]. Grouping chemicals into assessment groups based on biological and toxicological properties is preferred because this hazard-driven criterion provides the most scientifically robust and least uncertain basis for cumulative risk assessment [43]. This grouping can be further refined to reduce uncertainty, as shown by Nielsen et al. [44] in the identification of cumulative assessment groups (CAGs) for pesticide exposure. This refinement included a tiered approach for the establishment of four CAGs levels, where CAG1 active substances were grouped at the effect on the target tissue/organ level, CAG2 at the specific phenomenological effect level, CAG3 at the MoA level, and CAG4 at the mechanism of action level [44].

While HBGVs are usually defined based on the most critical or the most sensitive endpoint effect, mycotoxins have many additional and often entwined endpoint health effects. These effects, although not critical, could be of importance when considering combined exposure and forming common assessment groups. While not all these effects have sufficient evidence to be considered as established endpoints, they must be considered, whether they are observed in vitro, in animal studies, or in epidemiologic studies.

Across different mycotoxins, the most consistently reported effects include developmental and reproductive toxicity, carcinogenicity, immunotoxicity, and organ-specific toxicity targeting primarily the liver and kidneys. Apart from the genotoxic and carcinogenic critical effects of AFs, they can cause a range of additional effects after both short-term and prolonged exposure. These effects include inhibition of normal growth, liver and kidney damage, alterations in the intestinal microbiota, effects on reproductive and developmental parameters (e.g., brain development, low birth weight), immunosuppression, stunted child growth, induction of oxidative stress, and effects on steroid hormone homeostasis [13]. Next to nephrotoxicity and carcinogenicity, OTA is linked to immunotoxicity, neurotoxicity, developmental effects, bladder or hepatocellular cancer, induction of oxidative stress, disruption of protein production, lipid peroxidation, calcium metabolism, mitochondrial respiration, and sugar metabolism [14,45]. While not acutely potent in humans, FUMs are linked to kidney toxicity and eventual development of liver and kidney tumours, embryotoxicity, esophageal cancer, growth impairment, inhibition of ceramide synthases and neural tube defects [36]. DON is linked with reduced body weight gain, diarrhea, hematological disturbances, immunotoxicity, developmental and reproductive toxicity (reduced fertility, embryotoxicity, skeletal abnormalities, effects on body weight), oxidative stress, nutrient malabsorption, endocrine disruption, inhibited protein synthesis and even hepatoxicity [38,46]. T-2 and HT-2 toxins have anorectic effects upon short-term exposure, cause haematotoxicity and myelotoxicity, and inhibit protein synthesis or cell viability [47]. Apart from its oestrogenicity and related effects, ZEA induces genotoxicity and liver lesions accompanied by cancer development; it is immunotoxic and nephrotoxic, damages intestinal health, interferes with endocrine stability, and may induce retinopathy and cataracts [48]. Some studies indicate that PAT induces carcinogenicity, mutagenicity, teratogenicity, and neurotoxicity effects [45]. EAs’ exposure induces signs of neurotoxicity, effects on the reproductive process, and embryotoxicity [18]. Next to the nephrotoxicity, CIT affects other target organs such as the liver and bone marrow [49].

When assessing the combined effect of multiple chemicals within a component-based approach and grouping of chemicals into common assessment groups, the use of dose addition is a default assumption of the combined effect, meaning that every contaminant theoretically contributes to the cumulative effect proportionally to its dose and individual potency [24]. Dose addition typically gives the most conservative prediction, especially at low doses, and therefore this approach is preferred in decision-making processes [24,50,51]. Dose addition, or Loewe’s additivity model, assumes that components behave as dilutions of one another and share a common MoA [51,52]. While it is preferable to apply the dose addition model and define assessment groups based on a common MoA, a more pragmatic approach is commonly accepted. Current consensus holds that chemicals acting through different MoA, but leading to the same adverse outcome, may be grouped and assumed to produce combined effects according to the dose addition model, even when the underlying MoA is not fully understood [53,54,55,56].

An example of creating common assessment groups using a common target organ is shown in the work of van den Brand et al. [57], where mycotoxins were grouped as follows: diacetoxyscirpenol (DAS), nivalenol (NIV), moniliformin, T2, and HT2 toxin were grouped as mycotoxins that are relevant for effects on the hematological system; CIT, FUMs, NIV, OTA, and PAT were categorized as mycotoxins of concern for effects on the kidney; and FUMs and ZEN were categorized as mycotoxins of concern for effects on the liver. Vejdovszky et al. [58,59] created common assessment groups for nephrotoxicity, neurotoxicity and pre- and neonatal development, where they included not only mycotoxins but also other food contaminants that can cause the same adverse health effects.

Emphasizing the importance of cumulative mycotoxin exposure assessments, biomonitoring studies prove that humans are indeed continually exposed to multiple mycotoxins. Battilani et al. [10] summarized data on multi-mycotoxin biomonitoring studies that showed different combinations of mycotoxins in human biological fluids. From the 27 studies reporting multi-mycotoxin exposure, the following different combinations were registered: two mycotoxins—OTA+CIT, AFM1+OTA, CIT+OTA, AFs+FUMs, and AFs+DON; three mycotoxins—AFM1+FUMs+OTA, DON+FUMs+ZEN, AFs+FUMs+OTA, AFM1+OTA+DON, and ALT+DON+ZEN; four mycotoxins—CIT+DON+OTA+ZEN; five mycotoxins—AFs+DON+FUMs+OTA+ZEN, CIT+DON+ENNs+T2+ZEN, DON+FUMs+NIV, and OTA+ZEN; six mycotoxins—AFs+DON+FUMs+NIV+OTA+ZEN and AFs+DON+FUMs+OTA+T2+ZEN; and seven mycotoxins—AFs+CIT+DON+ENNs+FUMs+OTA+ZEN, DAS+DON+FUSX+NEO+NIV+T+ZEN, and DAS+DON+FUSX+NEO+NIV+T2+ZEN (ALT—altenuene, ENNs—enniatins, FUSX—fusarenon-X, and NEO—neosolaniol).

As humans frequently consume a mixture of various mycotoxins, due to both co-occurrence and dietary diversity, all possible interactions of these mycotoxins are also clearly important when deriving possible assessment groups. These interactions fall into three types: antagonistic, additive, and synergistic. Interaction types are characterized by comparing the observed combined effect of substances with the effect expected from their individual actions: when the observed effect exceeds the expected value, it is classified as synergism, when it is lower, it is considered antagonism, and when the observed and expected effects are equal, it is defined as an additive interaction [60]. Examples of possible mycotoxin interactions are presented in Table 2.

Examples shown in Table 2 are just a fragment of the published combined effects of mycotoxins [60,67]. The type of interaction reported for mycotoxin combinations depends on numerous factors, in particular cell models or specific organisms and endpoints, substance types, and their doses and mixture ratios. Such interactions cannot be incorporated into a general risk assessment scheme, but must be treated on a case-by-case basis [51,68,69].

Despite the presence of evident interactions within chemical (mycotoxin) mixtures, dose addition remains the default and preferred model due to its pragmatic advantages and its ability to provide conservative predictions at low exposure levels. Synergistic and antagonistic interactions are difficult to model even in binary mixtures, and the inclusion of multiple substances, each with potentially different interaction profiles, further increases methodological complexity several fold. The key practical benefit of applying dose addition as the default approach is its mathematical simplicity, as it can be readily implemented by comparing exposure doses or concentrations with reference values derived from toxicity data (e.g., no-effect or effect concentrations), which are often available in public databases [24].

## 3. Exposure Assessment

Exposure assessment is the process of qualitatively and/or quantitatively evaluating the likely intake of biological, chemical, or physical agents from food and other relevant exposure sources [70]. Accurate estimates of exposure to mycotoxins are essential, and exposure assessment could be considered the most essential and practical part of the whole process, or at least the one with the highest overall impact if not conducted properly.

Based on food consumption and mycotoxin concentration data, the exposure calculation follows a simple mathematical equation:

(1) $$EDI=\frac{\Sigma \;(Ci*IRi)}{BW}$$

where EDI is the Estimated Daily Intake, C represents the concentration of the mycotoxin in a particular food item *i* (e.g., µg/kg), IR denotes the intake rate or daily consumption of that food *i* (e.g., kg/day), and BW refers to the body weight (kg). In the case of multiple food items, total exposure is calculated as the sum of the products of concentration and intake across all relevant foods, normalized by body weight. As an extension of the basic exposure equation, processing factors can be incorporated as coefficients to account for changes in mycotoxin concentrations resulting from food processing. This is particularly relevant when occurrence data are derived from earlier stages of the food chain (e.g., raw agricultural commodities), where processing may significantly alter contaminant levels before consumption.

A review of the available literature, even at a cursory level, reveals substantial variability in the approaches used to assess dietary exposure to mycotoxins regarding the sources of food consumption data (i.e., using Food Balance Sheets, national dietary surveys, raw data from individual-level dietary surveys, and summary statistics from the EFSA Comprehensive Food Consumption Database); the sources of occurrence data on mycotoxin concentrations in food (monitoring data, national ML, literature sources, global database data); the length of intake (acute or chronic exposure); the number of food items in the analysis (single food exposure and aggregated exposure); and the modelling approaches applied (deterministic or probabilistic). Without aiming to provide an in-depth analysis of the applied methodologies, it can be noted that each approach may be fit for purpose depending on the desired level of accuracy and/or refinement. Although some methods may be associated with higher levels of uncertainty than others, they can still represent valuable sources of information.

While various approaches to dietary exposure assessment to mycotoxins are employed, there is an evident trend toward the use of individual-level food consumption data and larger, more comprehensive occurrence datasets, with an increased number of food items included in the analysis. In addition, the application of probabilistic modelling approaches has become increasingly prevalent. Probabilistic models introduce more realism in the risk assessment as they apply probability distributions to variables in a risk equation to capture both variability (true heterogeneity in exposure) and uncertainty (lack of knowledge about parameters) [71]. Probabilistic methods play an important role in cumulative risk assessment, as they can potentially incorporate parameters not readily addressed by deterministic approaches, thereby increasing the robustness of the assessment and minimizing the likelihood of over- or underestimation. Examples of these parameters are negative and positive correlations in food consumption, where consumption of fish and meat is negatively correlated, and meat and vegetables are positively correlated [24]. The increasing application of probabilistic models can be attributed to the growing availability of software tools on the market, including both commercial and open-access solutions, as well as to the expanding body of published research that has contributed to advancing methodological knowledge in this field. Some of the most used software for the mycotoxins exposure modelling are @RISK, Oracle Crystal Ball, and R-environment [72,73,74,75].

This increased use of statistical software has also enabled the use of advanced statistical treatment of left-censored data (i.e., negative values or non-detects). In most cases, these are not real negative samples but are values below a limit of detection (LOD) or limit of quantification (LOQ), representing limits of analytical methods. The most common approach for dealing with this type of data, observed in both deterministic and probabilistic methods, is the substitution method. In the substitution method, non-detect values are replaced with 0 or LOD (LOQ)/2 and LOD (LOQ) values, which create lower, middle, and upper bounds of the exposure [5,76]. Although the substitution method is still commonly preferred, it is widely recognized as biased [76]. Therefore, applying statistical approaches, such as maximum likelihood estimation, to determine the distribution parameters of left-censored mycotoxin contamination data can offer a more accurate, unbiased, and realistic solution to the problem [56,72,77].

When performing cumulative mycotoxin risk assessment, the exposure assessment step can be used to apply dose addition modelling with the use of Relative Potency Factors (RPF). This approach employs a scaling factor, the RPF, whereby the concentrations (or doses) of mixture components are expressed relative to an index compound and subsequently summed [68]. In other words, the concentrations of all chemicals are expressed as an equivalent of the most potent chemical in the mixture and then summed. The RPF of each mycotoxin can be calculated by dividing the RP of the identified reference (index) compound (RP_index_, i.e., the most potent compound) by the RP of each mycotoxin (RP_i_) as follows [78]:

(2) $$RPF=\frac{RP_{index}}{RP_{i}}$$

Then, the combined concentration C_m_, expressed in terms of the index compound for n compounds, is:

(3) $$C_{m}=\sum_{i=1}^{n}\left(C_{i}*RPF_{i}\right)$$

where C_i_ is the concentration of the *ith* mixture component, and RPF1 = 1, since *i* = 1 indicates the index chemical [68].

For this approach to be fully applicable, the derivation of RPFs needs to be based on the assumption of the toxicological similarity of substances in the mixture (i.e., same endpoint), and if possible, the data should originate from similar experimental setups [57,68]. This RPF approach was used by EFSA in the opinion on ZEN and its modified forms, where modified forms were expressed as ZEN equivalents, enabling combined exposure assessment under the assumption of dose addition [41]. A modification of this potency-adjusted exposure calculation was used by Battilani et al. [10] in an exploratory risk assessment case study of combined exposure to multiple mycotoxins in food, where they analyzed two mycotoxin mixtures from *Fusarium* mycotoxins, namely DON, FBs and ZEN, and T2/HT2, DON and NIV. An additional example of this approach was seen in a tiered approach to a mycotoxin mixture risk assessment by van den Brand et al. [57].

## 4. Risk Characterization

In the final stage of risk assessment, risk characterization, the findings from the exposure assessment and hazard characterization are combined to assess the potential risk to human health in a form suitable for decision-making in risk management [70].

Most exposure (risk) assessments of nongenotoxic/noncarcinogenic mycotoxins (threshold mycotoxins) report the risk in simple terms of EDI not exceeding or exceeding the HBGV set for individual mycotoxins. This can be translated as the Hazard Quotient (HQ) approach, where HQ is obtained by dividing mycotoxin EDI and its HBGV, as follows:

(4) $$HQ=\frac{EDI}{HBGV}$$

When HQ is less than 1, the risk is low, whereas an HQ greater than 1 indicates a risk for adverse health effects.

For mycotoxins with clear genotoxic/carcinogenic properties (i.e., AFs and OTA), the focus is on the Margin of Exposure (MOE) approach, in which the MOE is obtained by dividing the RP of a certain mycotoxin by its EDI, as follows:

(5) $$MOE=\frac{RP}{EDI}$$

As an RP, EFSA recommends the use of the BMDL_10_ [79]. An MOE smaller than 10,000 indicates a higher level of concern, while a MOE larger than 10,000 suggests a lower level of concern [79]. Although the MOE approach is primarily applied to genotoxic and carcinogenic substances, it has also been used for non-genotoxic substances and endpoints, in which case a lower MOE cutoff value is considered appropriate (e.g., non-neoplastic effects of OTA and an MOE cutoff value of 200 [14]).

Examples of cumulative risk assessments for mycotoxins are relatively limited in the literature, and are mostly focused on groups of structurally related compounds, such as AFs, FUMs, and DON and its modified forms. These are typically assessed by simple dose addition, expressed as the sum of their concentrations (e.g., total AFs or total FUMs), assuming equal potency. Nevertheless, there are also studies addressing mixtures of different mycotoxins, as well as combinations of mycotoxins and other contaminants sharing common toxicological endpoints, providing examples of more complex mixture risk characterization [10,57,58,59,75,80,81].

Building on the HQ approach, the Hazard Index approach represents a relatively simple solution for risk characterization of multiple mycotoxin exposure, as it can be calculated as the sum of HQs for each of the substances *i* in the mixture [24,57,68], as follows:

(6) $$HI=\sum_{i=1}^{n}HQ_{i}$$

As with HQ, an HI greater than 1 indicates the presence of risk. This approach is often considered as a first, screening, and conservative step in cumulative risk assessment. It is based on HBGV, which are in most cases derived from different critical effects, which can lead to overestimation of risk [43,82]. Alternatively, the Point of Departure Index (PODI) can be used. PODI is like the HI, but it uses RPs (PoDs) for the specific endpoint effect instead of HBGVs [24,68]. While this is more specific than HI, it can underestimate risk as it does not include health protective uncertainty factors (UF) incorporated in HBGVs.

Representing cumulative risk, the MOET approach uses the reciprocal sum of the reciprocals of the MOEs or the reciprocal of the PODI for each of the substances *i* in the mixture [83]. However, in this case, MOE does not necessarily represent risk related to genotoxic/carcinogenic compounds [84]. It can be calculated as follows:

(7) $$\frac{1}{MOET}=\sum_{i=1}^{n}\frac{1}{{MOE}_{i}}=\frac{1}{{MOE}_{1}}+\frac{1}{{MOE}_{2}}+\cdots +\frac{1}{{MOE}_{n}}$$

There are no universally established criteria for defining an acceptable MOET value, and an MOET of 100 is usually considered to be the minimum acceptable cutoff value [84,85]. Alternatively, if the exposure is calculated using RPFs (Equations (2) and (3)), the MOET can be expressed as a ratio of the RP_index_ and potency-adjusted exposure [10,24].

An example of the use of HI and MOET to assess cumulative risk of mycotoxins is presented in the work of de Sá et al. [81]. To evaluate the risk that mycotoxins from cereal-based foods pose to infants and children, they used the HI approach to assess the non-carcinogenic cumulative risk of nine mycotoxins and the MOET approach to assess the risk of carcinogenic and genotoxic mycotoxins, namely AFs and OTA [81].

Proposed by Vejdovszky et al. [58], the modified Reference Point Index (mRPI) combines the advantages of the HI and the PODI. This approach combines the sensitivity of HBGVs regarding UFs and the direct consideration of the particular endpoint effect of the RP [58,59]. The mRPI is calculated as the sum of Reference Point Quotients (RPQs):

(8) $$mRPI=\sum_{i=1}^{n}RPQ_{i}$$

where RPQ represents a single substance *i* in the mixture, and it is calculated as:

(9) $$RPQ=\frac{EDI_{i}*UF_{i}}{RP_{i}}$$

As with HI and PODI, an mRPI less than 1 indicates low health risks. In the same study, the authors also developed a method for the derivation of UFs, if not already available, based on the decision tree and generally accepted conventions [58,86].

In the context of cumulative risk assessment, the appropriate regulatory response to identified risks remains an area of ongoing consideration. Responding is relatively straightforward in the context of single-substance risk assessment; however, when an elevated risk is identified through cumulative risk assessment, determining the most appropriate measures becomes more complex. In other words, the challenge lies in identifying how to effectively reduce exposure to chemical mixtures originating from multiple sources. While examples of cumulative risk assessment are increasingly numerous in the literature, from a regulatory point of view, cumulative risk assessment for substance mixtures conducted by the EFSA, particularly in relation to pesticide exposure, is probably the only true example of mixture assessment. This is because pesticides, although classified as plant protection products, represent a highly heterogeneous group with respect to chemical class, translocation within the plant, and transformation into metabolites or degradation products. EFSA has performed three cumulative risk assessments of pesticide residues: one considering two chronic effects on the thyroid system, one looking at two acute effects on the nervous system, and a third on acute craniofacial alterations [54,84,85]. For these assessments, the MOET approach was used; a MOET value of above 100 was generally considered protective for human health, and a MOET of 100 at the 99.9th percentile of exposure was established as the threshold for regulatory consideration. In most cases, this value was within health protective limits. The most concerning case of MOET under 100 at the 99.9th percentile was for toddlers and children, and the nervous system assessment. While it was noted that the main drivers of exposure were active substances for which corrective actions had already been implemented by Member States (e.g., lowering legal limits), an equally important observation was that exposure estimates at the 99.9th percentile were driven primarily by the presence of a single pesticide in a single food commodity, rather than by simultaneous exposure to multiple substances [87]. Similar results were observed in the assessment conducted by Vejdovszky et al. [59]. This implies that risk management measures may be easier to implement if the cumulative risk is primarily attributable to a single substance–commodity combination, rather than to complex co-exposure scenarios involving multiple chemicals and sources.

Hazard assessment data are based on animal models, which can show important differences in biokinetic activity and the metabolite pattern compared to humans [88]. For that reason, in silico approaches have been increasingly applied. Among these so-called new approach methodologies (NAMs), physiologically based pharmacokinetic (PBPK) models have shown potential to improve hazard assessment by removing inter-species differences, and at the same time, reducing the need for animal experiments in human risk assessment [89]. PBPK models predict the absorption, distribution, metabolism and excretion of a substance in an organism through a series of differential equations representing the underlying physiological processes [90]. These models have been proven to be efficient in translating in vitro toxicity data for different toxic endpoints to in vivo data used to define PoDs and HBGVs [91]. They have been used for hazard assessment of single mycotoxins [91,92], but they have also found their application in predicting interactions between different substances [93].

Regardless of whether a single or cumulative exposure is assessed, the presented risk indicators share a common limitation: they are not an ideal basis for risk ranking, as they are based on different critical health endpoint outcomes, making direct comparisons between hazards or mixtures intrinsically difficult. Risk ranking can enable governmental and regulatory organizations to prioritize resources for mitigating hazards and their anticipated public health impacts by using a common denominator [94]. While several methods of risk ranking exist, the Disability Adjusted Life Year approach is considered one that provides the most comprehensive perspective because it incorporates morbidity, mortality, and severity of the exposure, thus allowing comparisons between very different types of hazards [95]. Still, for food hazards, its full application is limited to the most toxic and characterized contaminants, such as heavy metals and environmental contaminants. Key challenges in applying this approach include establishing causal links between chemical exposure and subsequent disease or mortality, inadequate evidence to estimate the incidences of chronic disability, the occurrence of the specific endpoints from other causes, and the time-intensive nature and complexity of the associated mathematical modelling and analytical processes [95,96,97]. In the field of mycotoxin research, this approach has been applied almost entirely to the risk assessment of AF exposure. This is because the quantitative HCC risk approach is based on the carcinogenic potency of AFB1 resulting from synergistic hepato-carcinogenic effects of AFB1 and hepatitis B virus infection [98]. This approach allows estimation of the occurrence of possible HCC cases, which can then later be used in counterfactual analysis in which the current disease outcomes with current exposure are compared to the disease outcomes under an alternate exposure [99].

## 5. Conclusions

Mycotoxins remain a significant challenge for food safety due to their frequent occurrence, diverse toxicological effects and the fact that they cannot be effectively eliminated from the food. As individual compounds, mycotoxins are well-characterized from a toxicological perspective, and numerous risk assessments have been conducted at the global level, covering a wide range of foods. These assessments illustrate diverse approaches, both in terms of the data sources and the mathematical and statistical modelling applied for exposure assessment. Given the frequent occurrence of mycotoxins and the documented co-exposure to multiple mycotoxins, greater attention should be paid to cumulative risk assessment, not only for mycotoxins themselves but also for other co-occurring contaminants. While well-established methodological frameworks exist to support cumulative risk assessment of mycotoxins, additional efforts are needed to define common assessment groups considering the pleiotropic nature of the mycotoxins.

## Figures and Tables

**Table 1:** Major mycotoxins and their principal producers.

Mycotoxin	Main Producer Species	References
AFs	*Aspergillus flavus* *Aspergillus parasiticus*	[10,13]
OTA	*Penicillium verrucosum* *Aspergillus ochraceus*	[10,14]
FUMs	*Fusarium verticillioides* *F. fujikuroi*	[10,15]
Deoxynivalenol (DON)	*Fusarium culmorum* *Fusarium graminearum*	[10]
T-2/HT-2	*Fusarium sporotrichioides* *Fusarium langsethiae* *Fusarium poae*	[10]
ZEN	*Fusarium culmorum* *Fusarium graminearum,* *Fusarium verticillioides*	[10,16]
PAT	*Penicillium expansum* *Aspergillus clavatus,* *Byssoclamys nivea*	[17]
EAs	*Claviceps* spp.	[18]
CIT	*Penicillium* spp. *Aspergillus* spp. *Monascus* spp.	[19,20]

**Table 2:** In vitro assessment of mycotoxin interactions.

Mixture	Endpoint Effect	Combined Effect	Cellular Model	Reference
STE+OTA OTA+PAT STE+PAT STE+OTA+PAT	Cytotoxicity	Synergistic	3D spheroids (tumour Neuroblastoma and healthy Mesenchymal Stem Cells)	[61]
AFB1+DON AFB1+ZEN DON+ZEN AFB1+DON+ZEN	Cytotoxicity	HepG2: Synergistic (DON+ZEN, AFB1+DON+ZEN) Additive (AFB1+DON) Antagonistic (AFB1+ZEN) RAW 264.7: Synergistic (AFB1+DON, DON+ZEN, AFB1+DON+ZEN) Additive (AFB1+DON, DON+ZEN, AFB1+DON+ZEN) Antagonistic (AFB1 + ZEN)	HepG2, RAW 264.7 cells	[62]
AFB1+FB	Cytotoxicity	Additive and synergistic	HepG2 cells	[63]
AOH+DON DON+ZEA AOH+ZEA	Cytotoxicity	Additive (AOH+DON, DON+ZEA) Synergistic (AOH+ZEA)	THP-1 cels	[64]
DON+ZEN	Cytotoxicity	Antagonistic	HCT116 cells	[65]
ZEN+T-2 DON+ZEN DON+T-2 DON+FB1	Myelotoxicity	Additive (ZEN+T-2, DON+ZEN) Additive and synergistic (DON+T-2) Antagonistic (DON+FB1)	hCFU-GM cells	[66]

## Data Availability

No new data were created or analyzed in this study.

## Abbreviations

The following abbreviations are used in this manuscript:

AFs	Aflatoxins
FUMs	Fumonisins
OTA	Ochratoxin A
ZEN	Zearalenone
PAT	Patulin
EAs	Ergot alkaloids
CIT	Citrinin
DON	Deoxynivalenol
MLs	Maximum levels
EFSA	European Food Safety Authority
MoA	Mode of action
RP	Reference point
PoD	Point of departure
HBGV	Health based guidance value
TDI	Tolerable daily intake
TWI	Tolerable weekly intake
NOAEL	No-observed-adverse-effect level
BMD	Benchmark dose
LOAEL	Lowest-observed-adverse-effect-level
BMDL	Lower confidence bound of the BMD
HCC	Hepatocellular carcinoma
CONTAM	EFSA Panel on Contaminants in the Food Chain
JECFA	The Joint FAO/WHO Expert Committee on Food Additives
CAG	Cumulative assessment group
DAS	Diacetoxyscirpenol
NIV	Nivalenol
LOD	Limit of detection
LOQ	Limit of quantification
RPF	Relative potency factors
HQ	Hazard quotient
MOE	Margin of exposure
HI	Hazard index
UF	Uncertainty factors
mRPI	Modified reference point index
RPQ	Reference point quotient
NAMs	New approach methodologies
PBPK	Physiologically based pharmacokinetic
